# Impact of a Culturally Tailored Education Intervention for African-American and Appalachian Men in Ohio

**DOI:** 10.1007/s13187-021-01970-y

**Published:** 2021-02-27

**Authors:** Bryn Dougherty, James L. Fisher, Toyin Adeyanju, Electra Paskett

**Affiliations:** 1 College of Medicine, The Ohio State University, Ohio, USA; 2 Comprehensive Cancer Center, The Ohio State University, Ohio, USA; 3 College of Public Health, The Ohio State University, Ohio, USA; 4 College of Medicine, The Ohio State University, Ohio, USA

**Keywords:** Cancer, Health disparities, Education, Men’s health, African American, Appalachian

## Abstract

Men, particularly those of underserved groups, bear a disproportionate cancer burden. Knowledge about cancer and screening is associated with adherence to screening guidelines. However it is possible that a correlation exists between an individual’s education attainment and ability to gain knowledge from health education. Men were recruited from Ohio counties with significant cancer disparities and asked to participate in three education sessions. Measures included a baseline survey collecting demographic information and pre- and post-intervention knowledge assessments about each of the topics. Paired *t* tests were used to determine whether there were statistically significant changes in mean scores after the intervention. Repeated measures of variance (ANOVA) conducted through generalized linear models (GLM) were used to determine if scores varied significantly by educational attainment. Appalachian men, regardless of level of educational attainment, had significant increases in knowledge for all topics. African American men with at least some college education demonstrated significant increases in knowledge for all three topics, while those with no college education had significant increases for only two topics. College education had a significant effect on scores for one topic among the Appalachian men and all three topics among the African American men. The interaction between change in score and higher educational attainment was significant for only one topic among Appalachian men and no topics among African American men. Higher educational attainment was associated with greater increases in knowledge scores for only one topic among Appalachian men and no topics among African American men. Culturally tailored health educational interventions are a promising approach to reducing disparities in cancer screening and outcomes among men of underserved groups. While all groups demonstrated increases in mean knowledge scores after participating in the educational intervention, there was not a significant association between educational attainment and increases in knowledge scores. Future research is needed to explore additional approaches to delivering health education and increasing the knowledge of men with lower levels of educational attainment.

## Introduction

It is well documented that, when compared with women, men have worse health outcomes across a wide breadth of diseases and conditions. This can likely be attributed to the fact that men are less likely to seek help from healthcare professionals than women and are more likely to normalize or ignore symptoms [1]. Even when men do present to a provider for care, they are less likely to go to a primary care provider and more likely to go to an emergency department or urgent care [2]. One explanation for men’s reluctance to engage in proactive health behaviors is that seeking health care is often viewed as an innately feminine behavior and being ill compromises a man’s masculine status in society [1]. Regardless of the reasons, the implications for men’s health are serious.

A comparison of cancer data for men and women exemplifies the deleterious consequences of men’s health behaviors. Both the incidence and mortality of malignancy are higher in men, while women tend to present with earlier-stage, lower-grade, less-aggressive, and more often unifocal cancers than men [3]. This is particularly concerning when considering the fact that the three most common cancers in men and the ones with the highest mortality in the USA—prostate, lung, and colorectal—are amenable to screening, with early detection being shown to improve morbidity and mortality.

The 5-year survival rate for colorectal cancer (CRC) is 80–90% if the cancer is localized to the bowel wall but drops to 14% if there is metastasis at diagnosis [4]. Screening with prostate specific antigen (PSA) alone or in combination with digital rectal exams (DRE) allows for earlier detection of prostate cancer compared with no screening, but there are conflicting results about the ability of prostate cancer screening to reduce mortality [5]. The National Lung Cancer Screening Trial (NLST) demonstrated a 15–20% reduction in mortality for patients receiving low-dose helical CT scans compared with traditional chest X-rays [6]. These cancers are also amenable to lifestyle changes, including smoking cessation, improved diet, and increased physical activity. According to the American Cancer Society (ACS), approximately 42% of cancers diagnosed in 2018 were potentially avoidable and due to smoking or a combination of excess body weight, physical inactivity, excess alcohol consumption, and poor nutrition [7].

Disparities in cancer screening and outcomes also exist among men. An analysis of data from the National Health Interview Survey identified specific groups of men who were the least likely to report a recent colorectal or prostate cancer screening, including men with less than a high school education, those with an annual income less than 139% of the federal poverty level, those without a usual source of health care, uninsured men, and men who had not consulted a doctor in the past 12 months [8]. Men of minority racial groups are more likely to belong to these groups and thus bear a disproportionate cancer burden. According to data from State Cancer Profiles published by the National Cancer Institute (NCI) and Centers for Disease Control (CDC), African American men in the USA demonstrate consistently higher incidence and mortality rates for all-site cancers when compared with national averages [9]. This disparity persisted between the years 2000 and 2016 despite decreases within each population. Furthermore, the ACS estimates that African American men are more likely than any other racial group to have prostate cancer and are more than twice as likely to die of prostate cancer than white men [4].

A second group of men who suffer poor cancer outcomes due to low socioeconomic status is men residing in Appalachia. Appalachia is a largely rural region of the USA spanning 13 states that is home to over 25 million people [10]. The per capita income in Appalachia in 2007 was $29,274, a value 20% lower than the national average [11]. Likewise, only 23% of working-age adults in Appalachia have at least a bachelor’s degree, compared with the national average of 30% [12]. Over the past three decades, cancer mortality among Appalachians decreased by 14%; however the disparity in cancer mortality between Appalachia and the country overall grew from 1 percentage point to 10 percentage points higher than the national rate [13]. Among men in the USA, Appalachians have a nearly 25% higher lung cancer rate than the rest of the country [14]. These disparities cannot be explained by racial and ethnic differences because of the uniquely large portion of the Appalachian population that is non-Hispanic Caucasian, making the region less racially heterogeneous than the rest of the country. Consequently, most attempts to explain the persistent disparities in cancer outcomes seen in Appalachia point to correlates of low socioeconomic status, including obesity, smoking, low cancer screening, and low rates of health insurance [15].

The damaging effects of these disparities on the lives of African American and Appalachian men necessitate further research to evaluate potential solutions. One study analyzing cancer disparities among minority populations in Ohio highlights the need to develop and implement education regarding cancer and prevention and early detection behaviors, such as smoking cessation, healthy diet, physical activity, healthy weight, and HBV vaccination tailored for less-educated, lower-income minority males [16]. Another argues for the importance of health service and promotion programs that target underserved and minority males, citing that men’s premature morbidity and mortality costs the US economy an estimated $479 billion annually [17]. These recommendations are rooted in the understanding that increased knowledge is associated with improved adherence to cancer screening recommendations [18] and that those who are less educated are less likely to have this knowledge [19]. Various studies have demonstrated that African American men, who are at an increased risk for prostate cancer, are more likely to lack knowledge about prostate cancer than white men [20]. Those who have completed higher levels of education are more likely to participate in health and educational programs [21], and this may at least partially explain the relationship between educational attainment and health knowledge. However, it remains unclear what the relationship is, if one exists, between an individual’s educational attainment and ability to benefit and gain knowledge from targeted health education.

The Health Belief Model (HBM) suggests that the likelihood of an individual performing an action, such as getting a recommended cancer screening, is associated with six key factors: perceived susceptibility, perceived severity, perceived benefits, perceived barriers, cues to action, and self-efficacy [22]. For example, perceived barriers are negatively associated with a recent fecal occult blood test (FOBT) or sigmoidoscopy for CRC screening, while perceived benefits are positively associated with a recent sigmoidoscopy or colonoscopy [23]. Drawing on this knowledge and other research illustrating the association between the tenets of the HBM and cancer screening behaviors, the Men’s Health Education Series (MHES) was designed to increase knowledge of important health problems affecting men through the delivery of culturally tailored education sessions for African American men in Franklin County, Ohio, and men in Appalachian Ohio. Educational materials were intended to target participants’ estimation of their own risk for cancer, highlight the value of screening, address perceived barriers to screening, and increase participants’ confidence in talking to their healthcare provider about pursuing screening. This study aims to evaluate the impact of this culturally tailored educational intervention for African American and Appalachian men by characterizing the relationship between educational attainment and change in knowledge scores among men in each population group (i.e., African American or Appalachian).

## Purpose

The purpose of this study was to evaluate the impact of the Men’s Health Education Series, a culturally tailored education intervention for Appalachian and African American men, by characterizing the relationship between educational attainment and change in knowledge scores.

## Methods

### Participant Recruitment

This study uses data from the Men’s Health Education Series, a program designed to deliver culturally tailored cancer education to underserved men. Prior to its start, The Ohio State University Institutional Review Board approved the study, and informed consent was obtained from all participants. In Ohio counties with significant disparities in cancer incidence and mortality, participating sites were established through the OSU Comprehensive Cancer Center, Center for Cancer Health Equity, and the Appalachia Community Cancer Network and included both small community organizations and larger companies. A total of 115 Appalachian men and 111 African American men age 18 and older were recruited through their affiliation with these participating sites. While community sites did not directly enroll participants, they assisted in recruitment by sharing advertising materials (flyers, personal communications, radio and print advertisements, emails) with the men in their community or organization. Program staff were responsible for verifying the eligibility of each participant and ensuring that all men were employees of the business or affiliated with the community organization at which the education was taking place. Participants were also encouraged to bring a friend or family member to future sessions in order to increase the reach of the program.

### Study Measures

Prior to participating in the intervention, participants completed a paper survey requesting demographic information and health beliefs and behaviors. The following variables were included in the analysis and, in some cases, collapsed: current age (18–29, 30–39, 40–49, 50–59, 60+); race (White, Black/African American, Asian, Native Hawaiian/Pacific Islander, American Indian/Alaska Native, other); ethnicity (Hispanic or Latino, not Hispanic or Latino); annual household income ($0–$10,999, $11,000–$15,999, $16,000–$20,999, $21,000–$25,999, $26,000–$35,999, $36,000–$50,999, $51,000–$75,999, $76,000 or more, I’m not sure); marital status (married, living together, divorced, widowed, separated, never married); educational attainment (less than a high school diploma or GED, high school diploma or GED, two-year associate degree, some college or technical school training, college degree or higher); insurance status (yes, no, I’m not sure); and healthcare provider (yes, no, I’m not sure). Educational attainment was collapsed to no college education and at least some college education. Before each session, participants were also asked to complete an 8-item pre-intervention knowledge assessment specific to the session being presented. These were also in paper format and were collected before the education began.

The education included two culturally tailored series, the Appalachian Men’s Health Education Series and the African American Men’s Health Education Series that were created for use in this study. Each series included three sessions: colorectal and prostate cancer; lung cancer, tobacco cessation, and clinical trials; and nutrition and physical activity. The sessions were presented using either a PowerPoint presentation or flip chart (depending on the size of the group), an educational video, and an open-ended facilitated discussion. Each session was designed with adult learning principles in mind and intended to be approximately half presentation and half application and feedback. The content for the sessions was adapted for underserved Appalachian and African American adult men from the NCI’s early detection messages, using evidence-based strategies addressing motivations for and barriers to engaging in health behaviors. All educational materials were reviewed by experts for accuracy. Two focus groups were held to pilot the educational materials in the series and better tailor them to each population group. The African American Men’s Health Education Series was piloted at a focus group of African American men in Franklin county on April 16, 2011. The Appalachian Men’s Health Education Series was piloted at a focus group of Appalachian men in Guernsey county on April 21, 2011. The men provided feedback on the content, pictures, and overall program design and made recommendations as to what they would like to see included or removed.

At the end of the session, participants were given an 8-item post-intervention knowledge assessment identical to the pre-intervention knowledge assessment for that topic. They did not have access to these assessments during the education. Each item on all three knowledge assessments had three response options: true, false, and I’m not sure.

The statements on the colorectal and prostate cancer knowledge assessment were as follows: (1) cancer is an uncontrolled growth of cells in the body; (2) screening for colorectal cancer should begin at age 40 for all “average risk” individuals; (3) you can lower your risk of getting colorectal cancer by increasing physical activity, avoiding tobacco, and eating a healthy, balanced diet (a risk factor is something that increases the chance of developing a disease); (4) a change in bowel habits is not a common symptom of colorectal cancer; (5) screening is checking for health problems after you have symptoms; (6) African Americans have a higher chance of developing prostate cancer; (7) urinary problems such as not being able to pass urine or having a hard time starting or stopping the urine flow may be symptoms for prostate cancer; and (8) it is important to talk with your healthcare provider about your risk of prostate cancer and your need for screening tests.

The statements on the lung cancer, tobacco cessation, and clinical trials knowledge assessment were as follows: (1) second hand smoke (smoke given off by a burning tobacco product) can cause lung cancer in nonsmoking adults; (2) lung cancer is the leading cause of cancer death among both men and women in the USA; (3) the best way to prevent lung cancer is to not smoke; (4) smokeless tobacco products are a safe substitute to smoking; (5) it is never too late to benefit from quitting smoking; (6) clinical trials are research studies that involve people and test new ways to prevent, find, diagnose, or treat diseases; (7) only people who have cancer can participate in a clinical trial; and (8) many safety procedures are in place to protect people who take part in clinical trials before and during the study.

The statements on the nutrition and cancer prevention knowledge assessment were as follows: (1) your nutritional needs depend only on your weight and height; (2) reading the labels on your food packages is important; (3) you do not have to make huge changes in your diet to be healthier; (4) the first thing to look at when reading a food label is the serving size and how many servings are in the package; (5) adults should be physically active for 30 min each day; (6) regular physical activity can help increase your body’s ability to fight illness, increase blood flow, and help you in sleeping well; (7) there is no need to do muscle strengthening activities 2 days a week if you do physical activity during the week (muscle strengthening activity is an activity that improves muscle strength by slowly increasing the ability to resist force through the use of free weights, machines, or the person’s own body weight); and (8) for a man, a waist circumference (waist size) over 40 inches places you at a greater risk for developing conditions such as Type 2 Diabetes, high cholesterol, high blood pressure, and heart disease.

Depending on the site, some men participated in all three sessions at one time, whereas others participated in only one at a time. If the men were participating in multiple sessions at once, the post-intervention knowledge assessment was administered after each individual session before proceeding to the next so that there were no delays between education and assessment. The men were also asked to complete a program evaluation at the end of each session. In appreciation for their time, the participants received a $10 store gift certificate for returning the baseline survey and $5 store gift certificates for each session they attended (maximum amount $15).

### Data Analyses

The analysis evaluated the changes in participant knowledge related to prevention and early detection of colon, lung, and prostate cancers and clinical trials as measured by pre- and post-intervention knowledge assessments. The men were each assigned a unique participant identification number (PID) that was used for identification throughout the database. These PIDs were used on all surveys and knowledge assessments to ensure all measures were matched accordingly. Correct responses were summed to produce a score for each knowledge assessment. Some participants did not respond to every question; non-responses were deemed incorrect. Participants with non-responses for more than half (4) of pre-test or post-test questions were not included in analyses. Therefore, it was possible for one participant to not be included in one, two, or all three assessments. Seven waves of participation have occurred, and, for pre-intervention and post-intervention results, these waves were analyzed together. Small sample sizes for several waves prevented analyzing the results according to wave. Paired (matched) *t* tests were the preferred test to determine whether there were statistically significant differences between pre- and post-intervention scores, for Appalachian and African American men, separately, and for varying levels of educational attainment separately. However, scores were not normally distributed (with a preponderance of negative or left skewing as the result of more participants with correct responses), and, as a result, the Wilcoxon signed-rank test was used to determine if there were statistically significant changes in pre-test, post-test scores. To determine whether pre-intervention scores and post-intervention scores varied significantly according to educational attainment among Appalachian men and African American men separately, scores were transformed by taking the square root of reflected values. Repeated measures were examined through general linear models (GLM). Statistical analyses were conducted using SPSS version 26. Hypothesis tests were two-sided, and alpha was set to 0.05 for statistical tests.

## Results

### Participant Characteristics

Descriptive characteristics of the sample population are presented in Table 1, stratified by educational attainment and population group. Among 167 Appalachian men, 79 (47.3%) had no college education and 88 (52.7%) had at least some college education. Of the Appalachian men with no college education, 76 (93.8%) were white, 75 (95.0%) were not Hispanic or Latino, 51 (64.6%) were married, 58 (73.4%) were age 40 or older, and 44 (55.7%) reported a household income of $50,000 or less. The majority of these men rated their health as good (*n* = 41, 51.9%), reported having some form of health insurance (*n* = 63, 81.0%) and reported having a healthcare provider (*n* = 52, 65.8%). Of the Appalachian men with at least some college education, 85 (96.6%) were white, 88 (100.0%) were not Hispanic or Latino, 55 (62.5%) were married, 63 (71.6%) were age 40 or older, and only 25 (28.4%) reported a household income of $50,000 or less. These men also most commonly rated their health as good (*n* = 52, 59.1%), reported having some form of health insurance (*n* = 83, 94.3%), and reported having a healthcare provider (n=67, 76.1%).

**Table 1:** Characteristics of Men’s Health Education Series participants, stratified by educational attainment and population group (n=341).

Variable	No college	At least some college		
	Appalachian (*n* = 79), n (%)	African American (n=77), n (%)	Appalachian (*n* = 88), n (%)	African American (*n* = 97), n (%)
*Demographics*				
Age category				
18–29 years	8 (10.1%)	10 (13.0%)	11 (12.5%)	6 (6.2%)
30–39 years	13 (16.5%)	3 (3.9%)	14 (15.9%)	4 (4.1%)
40–49 years	19 (24.1%)	18 (23.4%)	23 (26.1%)	17 (17.5%)
50–59 years	19 (24.1%)	15 (19.5%)	19 (21.6%)	34 (35.1%)
60+ years	20 (25.3%)	31 (40.3%)	21 (23.9%)	36 (37.1%)
Ethnicity				
Hispanic or Latino	2 (2.5%)	0 (0.0%)	0 (0.0%)	1 (1.0%)
Not Hispanic or Latino	75 (95.0%)	72 (93.5%)	88 (100.0%)	95 (97.9%)
Race				
White	76 (93.8%)	10 (12.8%)	85 (96.6%)	4 (4.1%)
Non-white	5 (6.2%)	68 (87.2%)	3 (3.4%)	93 (95.9%)
Marital Status				
Married	51 (64.6%)	33 (42.9%)	55 (62.5%)	61 (62.9%)
Living Together	4 (5.1%)	1 (1.3%)	2 (2.3%)	3 (3.1%)
Divorced	10 (12.7%)	11 (14.3%)	11 (12.5%)	16 (16.5%)
Widowed	1 (1.3%)	3 (3.9%)	2 (2.3%)	3 (3.1%)
Separated	2 (2.5%)	4 (5.2%)	2 (2.3%)	2 (2.1%)
Never Married	6 (7.6%)	25 (32.5%)	15 (17.1%)	10 (10.3%)
Household Income				
$0–$10,999	4 (5.1%)	16 (20.8%)	5 (5.7%)	6 (6.2%)
$11,000–$15,000	3 (3.8%)	6 (7.8%)	1 (1.1%)	3 (3.1%)
$16,000–$20,000	3 (3.8%)	3 (3.9%)	1 (1.1%)	1 (1.0%)
$21,000–$25,000	4 (5.1%)	3 (3.9%)	2 (2.3%)	5 (5.2%)
$26,000–$35,000	6 (7.6%)	5 (6.5%)	7 (8.0%)	9 (9.3%)
$36,000–$50,000	24 (30.4%)	19 (24.7%)	9 (10.2%)	20 (20.6%)
$51,000–$75,000	12 (15.2%)	9 (11.7%)	23 (26.1%)	15 (15.5%)
$76,000 or more	14 (17.7%)	2 (2.6%)	37 (42.1%)	31 (32.0%)
Not sure	4 (5.1%)	10 (13.0%)	0 (0.0%)	3 (3.1%)
*Health characteristics and behaviors*				
Health rating				
Excellent	4 (5.1%)	7 (9.1%)	14 (15.9%)	11 (11.3%)
Good	41 (51.9%)	39 (50.7%)	52 (59.1%)	58 (59.8%)
Fair	28 (35.4%)	28 (36.4%)	19 (21.6%)	22 (22.7%)
Poor	2 (2.5%)	3 (3.9%)	1 (1.1%)	3 (3.1%)
Not Sure	0 (0.0%)	0 (0.0%)	1 (1.1%)	1 (1.0%)
Any health insurance				
Yes	64 (81.0%)	62 (80.5%)	83 (94.3%)	87 (89.7%)
No	4 (5.1%)	10 (13.0%)	4 (4.6%)	9 (9.3%)
Not Sure	2 (2.5%)	5 (6.5%)	0 (0.0%)	1 (1.0%)
Any healthcare provider				
Yes	52 (65.8%)	59 (76.6%)	67 (76.1%)	79 (81.4%)
No	21 (26.6%)	14 (18.2%)	19 (21.6%)	15 (15.5%)
Not sure	2 (2.5%)	4 (5.2%)	1 (1.1%)	3 (3.1%)

Not all variables total 341 due to missing data

Among 156 African American men, 77 (49.4%) had no college education and 97 (62.2%) had at least some college education. Of the African American men with no college education, 72 (93.5%) were not Hispanic or Latino, 33 (42.9%) were married, 64 (83.1%) were age 40 or older, and 52 (67.5%) reported a household income of $50,000 or less. The majority of these men rated their health as good (*n* = 39, 50.7%), reported having some form of health insurance (*n* = 62, 80.5%), and reported having a health care provider (*n* = 56, 76.6%). Of the African American men with at least some college education, 95 (97.9%) were not Hispanic or Latino, 61 (62.9%) were married, 87 (89.7%) were age 40 or older, and only 44 (45.4%) reported a household income of $50,000 or less. These men mostly rated their health as good (*n* = 58, 59.8%), reported having some form of health insurance (*n* = 87, 89.7%), and reported having a healthcare provider (*n* = 79, 81.4%).

### Knowledge Scores

**Fig. 1:**
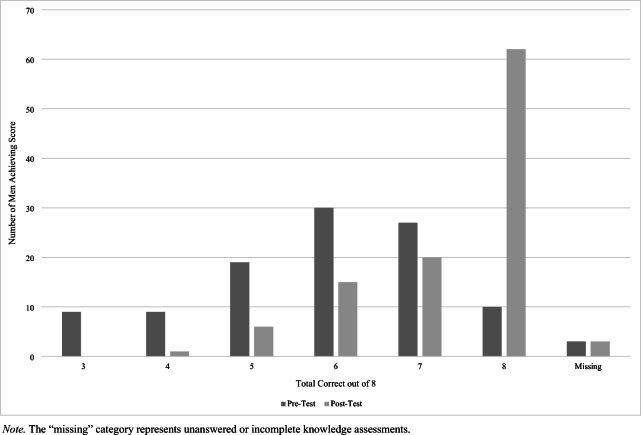
Distribution of scores on colorectal and prostate cancer pre- and post-intervention knowledge assessments among Appalachian men. The “missing” category represents unanswered or incomplete knowledge assessments

**Fig. 2:**
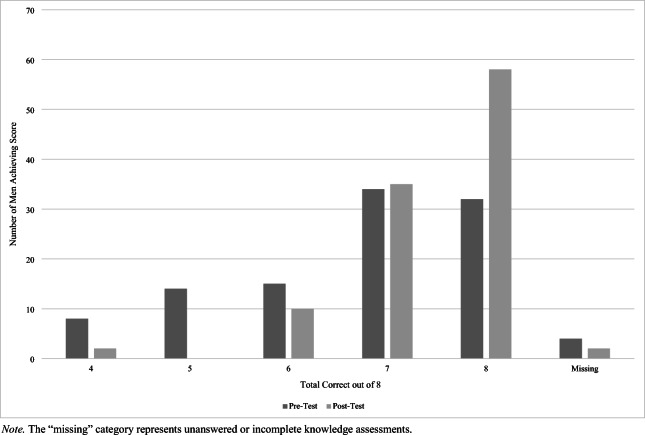
Distribution of scores on nutrition and cancer prevention pre- and post-intervention knowledge assessments among Appalachian men. The “missing” category represents unanswered or incomplete knowledge assessments

**Fig. 3:**
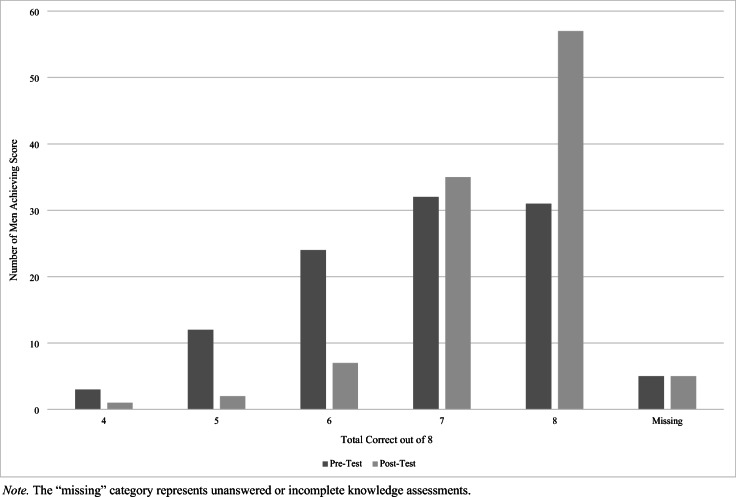
Distribution of scores on lung cancer, tobacco cessation, and clinical trials pre- and post-intervention knowledge assessments among Appalachian men. The “missing” category represents unanswered or incomplete knowledge assessments

**Fig. 4:**
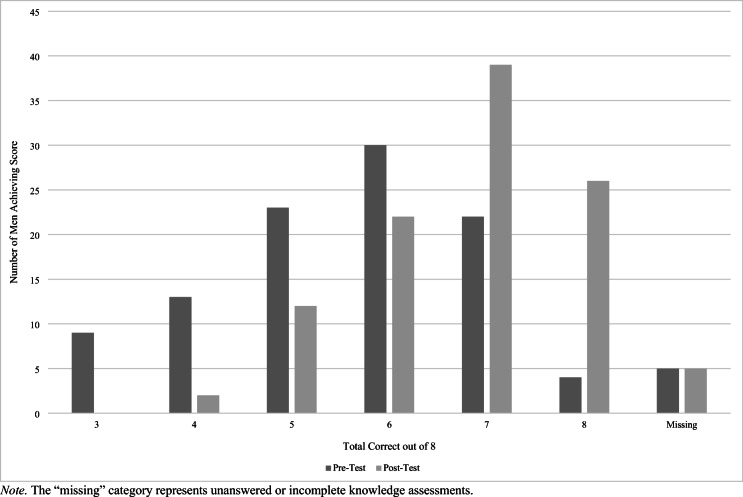
Distribution of scores on colorectal and prostate cancer pre- and post-intervention knowledge assessments among African American men. The “missing” category represents unanswered or incomplete knowledge assessments

**Fig. 5:**
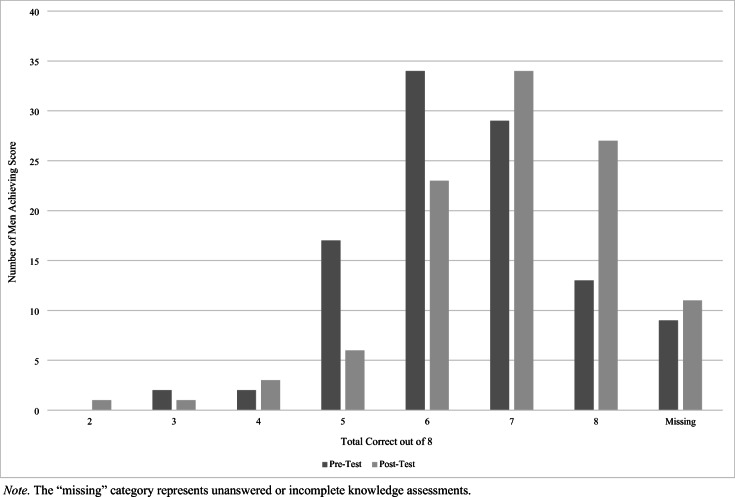
Distribution of scores on nutrition and cancer prevention pre- and post-intervention knowledge assessments among African American men. The “missing” category represents unanswered or incomplete knowledge assessments

**Fig. 6:**
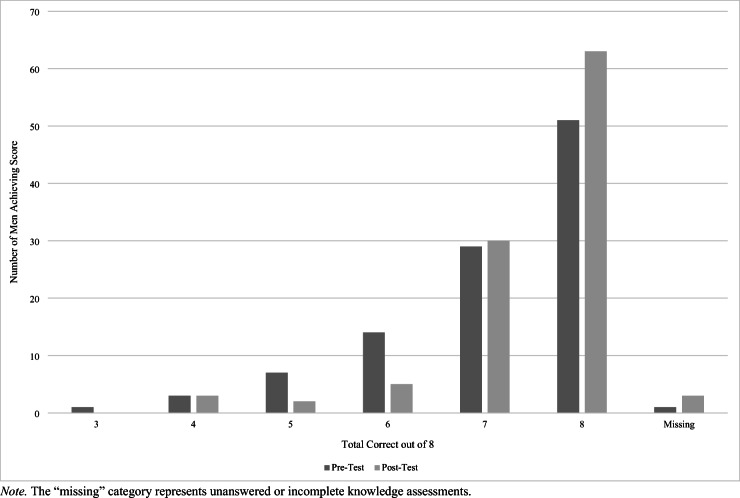
Distribution of scores on lung cancer, tobacco cessation, and clinical trials pre- and post-intervention knowledge assessments among African American men. The “missing” category represents unanswered or incomplete knowledge assessments

115 Appalachian men and 111 African American men completed knowledge assessments; however, there were inconsistent missing responses for each question on each assessment, resulting in varying sample sizes. The distribution of scores for each topic on pre- and post-intervention knowledge assessments in each population group are represented in Figs. 1, 2, 3, 4, 5 and 6 of the appendix. Tables 2 and 3 present mean scores for 8-item pre- and post-intervention knowledge assessments in Appalachian and African American men, respectively. Appalachian men with no college education demonstrated significant increases in knowledge for the colorectal and prostate cancer (*p* < 0.001); lung cancer, tobacco cessation, and clinical trials (*p* < 0.001); and nutrition and cancer prevention (*p* < 0.001) topics. Similarly, Appalachian men with at least some college education demonstrated significant increases in knowledge for the colorectal and prostate cancer (*p* < 0.001); lung cancer, tobacco cessation, and clinical trials (*p* < 0.001); and nutrition and cancer prevention (*p* < 0.001) topics. African American men with no college education had significant increases in knowledge for colorectal and prostate cancer (*p* < 0.001) and nutrition and cancer prevention (*p* = 0.027), while those with at least some college education had significant increases for colorectal and prostate cancer (*p* < 0.001); lung cancer, tobacco cessation, and clinical trials (*p* = 0.008); and nutrition and cancer prevention (*p* = 0.006).

**Table 2:** Mean scores for 8-item pre- and post-educational intervention knowledge assessments in Appalachian men, stratified and compared by educational attainment

	No college (*n* = 47)				At least some college (*n* = 58)				
Test	Pre-intervention score (median) [95% CI]	Post-intervention score (median) [95% CI]	*P*	Test	Pre-intervention score (median) [95% CI]	Post-intervention score (median) [95% CI]	*P*	*P* for at least some college	*P* for interaction between change score and at least some college
Colorectal and prostate cancer	5.8 (6) [5.4–6.2]	6.9 (7) [6.6–7.2]	< 0.001	Colorectal and prostate cancer	5.9 (6) [5.3–6.1]	7.5 (8) [7.2–7.8]	< 0.001	0.038	0.008
Lung cancer and tobacco cessation	6.7 (7) [6.3–7.1]	7.3 (8) [7.1–7.6]	< 0.001	Lung cancer and tobacco cessation	6.8 (7) [6.8–7.1]	7.5 (8) [7.3–7.7]	< 0.001	0.593	0.651
Nutrition and cancer prevention	6.8 (7) [6.4–7.2]	7.4 (8) [7.1–7.6]	< 0.001	Nutrition and cancer prevention	6.6 (7) [6.2–6.9]	7.4 (8) [7.2–7.6]	< 0.001	0.578	0.334

**Table 3:** Mean scores for 8-item pre- and post-educational intervention knowledge assessments in African American men, stratified and compared by educational attainment

	No college (*n* = 51)				At least some college (*n* = 52)				
Test	Pre-intervention score (median) [95% CI]	Post-intervention score (median) [95% CI]	*P*	Test	Pre-intervention score (median) [95% CI]	Post-intervention score (median) [95% CI]	*P*	*P* for at least some college	*P* for interaction between change score and at least some college
Colorectal and prostate cancer	5.3 (5) [4.9–5.7]	6.5 (7) [6.2-6.8]	< 0.001	Colorectal and prostate cancer	5.8 (6) [5.5–6.1]	7.0 (7) [6.8–7.3]	< 0.001	0.009	0.864
Lung cancer and tobacco cessation	7.0 (7) [6.6–7.3]	7.2 (8) [6.9-7.5]	0.264	Lung cancer and tobacco cessation	7.2 (7.5) [6.9–7.5]	7.7 (8) [7.5–7.8]	0.008	0.058	0.278
Nutrition and cancer prevention	6.0 (6) [5.8–6.3]	6.5 (7) [6.2-6.9]	0.027	Nutrition and cancer prevention	6.5 (7) [6.2–6.9]	6.9 (7) [6.5–7.3]	0.006	0.005	0.941

Tables 2 and 3 also demonstrate the effects of having at least some college education on scores. Higher educational attainment had a significant effect for the colorectal and prostate cancer topic (*p* = 0.038) among Appalachian men and a marginal to significant effect for colorectal and prostate cancer (*p* = 0.009); lung cancer, tobacco cessation, and clinical trials (*p* = 0.058); and nutrition and cancer prevention (*p* = 0.006) among African American men. The interaction between having at least some college education and changes in scores between pre- and post-intervention assessments differed slightly between the two population groups. Among Appalachian men, the interaction was significant for colorectal and prostate cancer (*p* = 0.008) only. However, among African American men, the interaction was not significant for any of the three topics.

## Discussion

Given the disparities in cancer screening and outcomes that afflict underserved populations, it is important to understand fully the impact of public health interventions and how various aspects of socioeconomic status influence an individual’s ability to benefit from them. Therefore, the objective of this study was to characterize the relationship between educational attainment and increase in knowledge scores among Appalachian and African American men in Ohio after their participation in the culturally tailored Men’s Health Education Series. The mean scores for those with at least some college education were the same or higher than for those with no college education for pre- and post-intervention assessments in all three topics in both population groups, with the exception of the nutrition and cancer prevention pre-intervention assessment in Appalachian men. Appalachian men, regardless of level of educational attainment, demonstrated significant increases in knowledge for all three topics. However, African American men with no college education demonstrated a significant increase for only one topic, while those with at least some college education demonstrated significant increases for all three. Higher educational attainment had a significant effect on scores for only the colorectal and prostate cancer topic among Appalachian men and all three topics among African American men. The more consistently significant association between higher educational attainment and higher scores among African American men may be attributed to the fact that African American men with no college education had a significant increase in knowledge for only one topic, while Appalachian men with no college education had significant increases for all three. The interaction between higher educational attainment and change in knowledge scores was significant for only colorectal and prostate cancer among Appalachian men and no topics among African American men.

Even though the educational materials were culturally tailored and piloted within the study populations to ensure that they were easy to understand, equivalent increases in knowledge were not seen across the two educational groups in both populations. While both groups of Appalachian men had significant knowledge increases for all topics, there was a disparity between African American men of different levels of educational attainment. One possible explanation is that those who have completed more education have an increased capacity for knowledge acquisition. However, it is also possible that the educational materials were more successfully tailored to the Appalachian population than to the African American population.

These findings are consistent with the literature, as multiple studies have documented the relationship between educational attainment and increased baseline health knowledge [24]. Additionally, studies have linked low health literacy, a correlate of lower educational attainment, with less health knowledge [25, 26]. Furthermore, one study evaluating the relationship between educational attainment and learning efficacy unrelated to health topics demonstrated that post-secondary educational attainment was associated with greater improvements on a series of cognitive assessments after a training intervention [27]. While our results support the findings of the aforementioned studies, we believe ours is the first study to establish a relationship between educational attainment and changes in knowledge in the context of a culturally tailored health education intervention. These findings contribute to the growing literature on health disparities among men and socioeconomic predictors of health knowledge and outcomes.

Strengths of this study include the focus on underserved men from areas with documented disparities in cancer outcomes and the involvement of members of the community to pilot the study materials. One limitation of this study is the relatively small convenience sample of 226 men. The men were recruited through their affiliations with certain community members or organizations. While this method helped ensure that the education sites were easily accessible to the participants, it is possible that some selection bias resulted in the participants having higher baseline knowledge levels or increased desire to learn about issues affecting their health than if the participants had been recruited using a population-based approach. Second, the participants’ changes in knowledge were only assessed immediately after participation in the education sessions. Additional knowledge assessments later on could elucidate how well the men from each population group were able to retain the information they learned. Furthermore, this study only included two underserved populations of men in Ohio, Appalachians, and African Americans. For these reasons, these findings may not be as generalizable to larger populations. Additionally, all of the demographic information, including income, educational attainment, insurance status, and having a healthcare provider, was self-reported by participants without validation.

Future efforts should aim to evaluate additional approaches to conducting health education interventions in underserved groups. While the mean scores for each knowledge assessment in every group improved after the educational intervention, the Appalachian and African American men with at least some college education did not appear to benefit more from the education, as evidenced by the absence of significant associations between educational attainment and increase in knowledge scores for most topics in each population group. Therefore, future studies should expand upon this work by determining methods to best tailor health education to underserved groups. Similar studies should also be conducted among different underserved populations to test the generalizability of these findings. These future investigations should consider incorporating follow-up surveys to assess if increases in knowledge are sustainable and if there are any changes in the men’s health behaviors. Conducting these follow-up assessments around 12 months from participation in the study would give participants ample time to schedule appointments with their providers and complete cancer screenings. This additional information would speak to the efficacy of this educational series as a way to improve cancer screening rates in underserved males. Additionally, further research could elucidate the ways in which community-based culturally tailored health education can be adapted so that healthcare providers can deliver it in office or hospital settings.

Culturally tailored health educational interventions are a promising approach to reducing disparities in cancer screening and outcomes among men of underserved groups. While all groups demonstrated increases in mean knowledge scores after participating in the educational intervention, there was not a significant association between educational attainment and increases in knowledge scores. Future research is needed to explore additional approaches to delivering health education and increasing the knowledge of men with lower levels of educational attainment.
